# On the Medical Use of Alkalis in Several Diseases
*We have already mentioned in our Journal, Vol. iii. p. 572, Dr. Stutz's Method of curing the Tetanus by means of Alkalis, &c.


**Published:** 1801-05

**Authors:** 


### *On the Medical Use of Alkalis in several Diseases.**

### *By* Dr. STUTZ.

[ Extracted from Hufeland's Journal, Vol. X. No. 4. ]

AMONGST the multitude of remedies which are exposed to the vicissitude of custom and fashion, none seem to have been so little regarded as the alkaline salts or alkalis. It is indeed surprising how much the use of alkalis, and particularly of the fixed one, has been hitherto confined by medical men. Salia media, acids, were frequently employed in different diseases from the earliest times of Medicine, whereas the alkalis were almost utterly neglected. Although the volatile alkali has been sometimes given in different disorders with much avail, yet the pure fixed alkali, mineral and vegetable, was but rarely or not at all used. Thus it has been only empirically recommended in the acor primarum viarum, in the rickets, where ABILDGAARD thinks it of avail in combination with bark and Rubia tinctorum; in dysenteries, in dropsy, by STORK, ABERTAUFFER, and THEDEN, the last of whom gave it with wine; in all which diseases however it was only employed by a few physicians, and at the same time mostly in combination with different other remedies, that it is impossible to say which of them proved efficacious.

It was reserved to our enlightened age, to point out the medical excellency of alkalis, and to draw the attention of medical

---

* We have already mentioned in our Journal, Vol. iii. p. 572, Dr. Stutz's Method of curing the Tetanus by means of Alkalis, &c.

medical men to a remedy, whose efficacy in the most dangerous affections appears to be inconteftible. The excitant powers of the fixed alkali were not at all, or very little known, till the ingenious naturalift Mr. HUMBOLDT firft hinted how efficacious a remedy it might prove; and till it was really employed in convulfions with great fuccefs by Dr. MICHAELIS and WIEDEMAN, but afterwards it feemed again to be laid afide. In October 1799, reading the experiments of Mr. HUMBOLDT on the influence which chemical fubftances have on excitability, and being led by farther reflexion and comparifon, I firft conceived the idea of employing the fixed alkali in one of the moft terrible difeafes, the Tetanus, which I had a frequent opportunity of obferving in the Imperial Chief Hofpital. The great fuccefs with which this new method of cure was crowned, prevailed on the Council of War at Vienna, that copies of my cafes, and the method of cure which I have defcribed, fhould be diftributed amongft the phyficians of the field hofpitals of the Imperial army, in order to put that method to a fair trial. As the alkalis, in combination with opium, proved fo highly ferviceable in one of the moft dangerous and mortal difeafes, I concluded farther, that their ufe might extend to other difeafes which originate in the fame proximate caufe. On this fubject my ideas are the following.—*Alkalis, mild as well as caustic, are useful in all asthenic and spasmodic diseases of the nervous and muscular fibre of whatever species and form.* All fpafmodic difeafes differ only with refpect to the individuals they attack, but agree in their internal character, which is common to all of them, that is to fay, *in the character of the debilitated organic fibre.* That thefe difeafes originate in a debility of the organic fibre, feems to be a matter of no doubt; for, elfe, how could a fibre, endowed with proper force and elafticity, be reduced by internal caufes to fuch irregular motions as in convulfions, or to fuch inactive contractions as in the tonic fpafms; it is certain that the debilitated organic fibre can only be affected in fuch a way. Excitant and corroborant remedies are therefore particularly indicated here, to remove that debility of the nervous and mufcular fibre; for which purpofe, I recommend the fixed vegetable alkali and opium, alternately given, and the cauftic alkali at the fame time externally applied. The excitant and corroborant properties of alkalis, though in fome meafure acknowledged in the volatile and cauftic alkali, was firft difcovered by the experiments of Mr. HUMBOLDT, on which my method of cure in Tetanus was built, and the fuccefs of which has proved the juftnefs of afferting that the alkalis belong to the excitant clafs of remedies.

The

The particular difeafes, however, in which alkalis may be ufed with hopes of the greateft fuccefs, are,

1. *The Tetanus and Trismus Traumaticus*, which I fucceeded in curing, in a fhort time, by externally applying the cauftic alkali in a hot bath, and by giving the mild one internally, particularly by alternately adminiftering opium at the fame time; upon which method of cure I fhall treat more fully in a peculiar effay.

2. Another difeafe, in which the alternate application of alkalis and opium has been of the greateft avail, are *the convulsions of pregnant women*, a difeafe equally dangerous. Although convulfions in children have been fuccefsfully removed by vegetable alkalis, according to the obfervations of Doctors MICHAELIS and WIEDEMANN, yet there was no certain method eftablifhed, and the alternate ufe of alkalis with opium, and their application in baths and fomentations was ftill unknown. Inftead of reafoning about this matter, I beg leave to relate the hiftory of a cafe communicated to me by the ingenious furgeon Profeffor BRUNINGHAUSEN, at Wurzburg.

" A healthy young lady, aged about twenty years, of a very mild temper but of an irritable conftitution, being pregnant for the firft time, complained after the feventh month of her pregnancy, (till which period fhe had always been very well) of a violent griping in the belly, without any evident caufe. A few hours after fhe felt a fpafmodic contraction of the left leg, which attempting to ftretch out, fhe was feized by the moft violent convulfions of the whole body, that lafted about ten minutes, and returned half an hour after. The phyfician who attended her, ordered her to be bled immediately, as the young woman was of a plethoric habit, her face extremely red and fwollen, and the pulfe ftrong and full; after which, clyfters with laudanum and afafœtida, fomentations of emollient herbs on the belly, and frictions on the joints with volatile liniment and laudanum were applied, and internally valerian with opium prefcribed; which remedy, however, fhe was not able to fwallow, on account of the locked ftate of the jaws, the fenfelefsnefs, and the utmoft difficulty of fwallowing. The intervals of the return of the convulfions increafed by degrees, but the fits became more violent. On the 18th of June I was called, partly in order to bring on her labour, if I thought proper; partly to make the Cæfarean operation, in cafe the patient fhould perifh in a fit of thefe convulfions, for the purpofe of faving at leaft the life of the child. Before my arrival the patient had already fuffered twenty-three of the moft violent fits, each of which threatened to kill her. The face became always red and fwollen fhortly before a fit came on, which, therefore, was was forefeen by thefe fymptoms; the eyes were, at the fame time, ftaring, and the pupils immoveable; and at a deeper infpiration, a murmuring noife was heard, and a bloody foam appeared at the mouth. All the mufcles of the extremities were in a continual viciffitude of rapid contraction and extenfion, and the face of the lady became horridly diftorted and blue. Each paroxyfm always terminated in about eight or ten minutes with the moft violent contractions of the extenfors, while the fnoring, fenfelefs, and comatofe ftate continued. The pulfe was frequent and hard. On touching the belly for a confiderable time, I remarked no motion of the child; and examining the os uteri, I found it ftill fo high that I could hardly reach it; it was foft, but fhut in fuch a manner that the point of my forefinger could not enter. Whilft I was thus examining, the patient relapfed again into a vehement convulfive paroxyfm; neverthelefs, I continued the examination for fome moments, purpofely to difcover whether the convulfions had any influence upon the orifice, which, however, was not the cafe, as it remained relaxed and immoveable; but the uterus was moft violently contracted, of which I convinced myfelf by touching the belly during the paroxyfm; it then became foft, and no fymptom of a labour appeared. Under fuch embarrafsing circumftances, there was not any indication for an artificial delivery; on the contrary, there was reafon to fear that on attempting to deliver her, the convulfions might be fo much increafed, as to deftroy the patient immediately. When we met again in the afternoon, the ftate of the patient was rather worfe; the convulfions had become more violent, and the paroxyfms brought on after rubbing in oil with opium. The ftate which fhe was in being fo very defperate, her relations defired me to defift from any further attempt to relieve her, as they thought fhe muft certainly die; and I fincerely confefs we were of the fame opinion. In this alarming fituation, I was put in mind of the method of cure which Dr. Stutz had fo fuccefsfully employed in three cafes of Tetanus; and thinking, that however different the external appearance of thofe difeafes might be, yet they agree in their nature and proximate caufe, I propofed the matter to my colleague, the attending phyfician, who readily adopted my idea. As there was no time to be loft, we inftantly prefcribed, ℞. Sal. tart. alc. dr. j. folv. in aq. diftillat. unc. iv. two table fpoons full to be taken every two hours, and in the intervals ten drops of laudanum. Doubting, however, of the patient being able to fwallow the medicine, we endeavoured to apply it as near to the uterus and lumbar nerves as we poffibly could. Half an ounce of Sal. tart. alcal. was accordingly prefcribed, of which two fcruples with a decoction of camomile flowers were ordered to be applied in a clyſter. We had wiſhed to put the patient into a hot bath; but ſhe being ſo far reduced, we were apprehenſive of her dying in it; I therefore recommended an application of the cauſtic alkali on the belly in fomentations, and one ounce of lapis cauſtic chirurgorum was accordingly diſſolved in one pint and a half of water, into which a cloth was dipped, and after it had been wrung out, put on the belly. I never ſaw a more ſtriking effect of any medicine than in this caſe. Two table ſpoons full of the medicine were given to her, which ſhe kept for a while in the mouth, but ſwallowed it at laſt. After the firſt clyſter and fomentation had been applied, a very extraordinary change was remarked. The eyes, which were before ſtaring, moved continually; the whole body, which lay before in an apoplectic ſtate, became reſtleſs; ſhe began to lift the arms, that had been quite lame, and to ſcratch the belly with her hand, becauſe an itching was probably occaſioned by the fomentations, as we imagined from a ſlight redneſs that was perceived on the belly. The paroxyſms, which before came on every quarter of an hour, remitted now for two hours; and after the repeated application of thoſe remedies, entirely ceaſed; the patient became quiet, recovered her ſenſes, and anſwered to ſeveral queſtions. At laſt ſhe began to complain of pains in the belly, which became afterwards true pains of labour, and were not at all interrupted by convulſions. About ſix o'clock in the evening ſhe was delivered of a child, which, though very weak and apparently dead, was with much trouble, reſtored to life within an hour. The placenta came out without difficulty; a ſound ſleep enſued; and we congratulated ourſelves upon this unexpected happy ſucceſs; when ſhe was, an hour after the delivery, ſuddenly attacked by a new paroxyſm, more violent than any ſhe had before experienced; and after the ſhort interval of ſix minutes, another, equally violent, ſucceeded. All ſeemed now to be loſt; we had, however, recourſe to the ſame remedies, and fortunately they did not forſake us. After a clyſter with one drachm of Sal. alcal. vegetab. had been applied, the fomentations renewed, and ſome table ſpoons full of the above ſolution adminiſtered, which remedies were ſome hours continued alternately with opium, ſhe fell aſleep again, and a copious perſpiration came on, after which the convulſive fits completely ceaſed. The lady was very weak, but recovered her ſtrength in a ſhort time. I abſtain from every remark upon this caſe, by which the great uſe of the above treatment ſeems to be evidently proved, and only add, that it ought to be remembered that, according to experience, convulſions in the laſt months months of pregnancy are moft dangerous affections, and almoft always prove fatal."

3. *In convulsions of every kind of adults as well as of children*, the alkalis are, according to my own experience and the obfervations of MICHAELIS, WIEDEMANN, &c. of very great fervice, particularly when alternately adminiftered with opium. In flight cafes of convulfions of children, a difeafe to which fo many fall a facrifice, the Sal. tartari, or the Oleum tartari per deliquium, is very ferviceable; but in a higher degree of organic debility, I employed volatile alkali, viz. the fprit. c. c. fuccinat. in a few drops in a proper vehicle, generally in Syrup. de cichor. cum Rheo. In clyfters, the fpirit of hartfhorn is likewife of avail in thefe cafes, in a greater or lefs dofe, according to circumftances. I have alfo had frequent opportunity of obferving the good effects of the fpirit of hartfhorn in the hooping cough, a difeafe that is certainly of a fpafmodic nature, upon which, however, I fhall treat more fully at another time, and only relate a cafe, by which the uncommon efficacy of alkalis with opium, in convulfions of adults, is moft evidently fhown.

[ To be continued. ]

